# Genomic analysis of *Escherichia coli*, *Enterobacter hormaechei*, and *Cupriavidus gilardii* isolated from *Cacatua alba*

**DOI:** 10.1007/s11259-026-11490-z

**Published:** 2026-08-29

**Authors:** Julia Penna de Andrade, Henrique da Silva Vieira, Camilla Faria Soares, Marcela Laryssa Oliveira Duarte, André Almeida Fernandes, Hannah Luiza Gonsalves Coelho, Victória Veiga Alves, Bertram Brenig, Vasco Ariston de Carvalho Azevedo, Flávia Figueira Aburjaile, Nelson Rodrigo da Silva Martins

**Affiliations:** 1 Departamento de medicina veterinária preventiva, Universidade Federal de Minas Gerais, Belo Horizonte, Brasil; 2 Institute of Veterinary Medicine, University Göttingen, Göttingen, 37077 Germany

**Keywords:** One Health, Pathogen emergence, Resistome, Psittacidae, Non-fermentative gram negative bacteria

## Abstract

The objective of this study was to perform genomic, phylogenomic, and phenotypic characterization of bacterial isolates from a captive *Cacatua alba* to investigate their antimicrobial resistance profiles, virulence-associated genes, and genomic relationships with publicly available strains. Bacterial isolates obtained from the lungs and encephalon were identified as *Escherichia coli* (AV037), *Enterobacter hormaechei* (AV038), and *Cupriavidus gilardii* (AV039). Comparative genomic analyses, phylogenomic reconstruction, and antimicrobial susceptibility testing (AST) were performed. Genomic analysis identified multiple antimicrobial resistance-associated genes in AV037, AV038, and AV039, with genes associated with efflux pumps, particularly those in the resistance-nodulation-cell division (RND) family, as the predominant resistance-associated mechanism identified. Most identified genes are intrinsic to the respective bacterial species and do not necessarily confer phenotypic antimicrobial resistance when present alone. Phenotypic testing confirmed multidrug-resistant (MDR) profiles, with resistance to β-lactams and aminoglycosides. Susceptibility to carbapenems was preserved in AV037 and AV038, whereas AV039 exhibited resistance to meropenem. Phylogenomic analysis demonstrated that AV037 and AV038 clustered with strains recovered from diverse hosts and geographic regions. In contrast, AV039 clustered predominantly with environmental and human-associated isolates, including strains harboring the *mcr-5* gene. These findings are consistent with previous reports describing the recovery of *C. gilardii* from both environmental and clinical sources. Overall, this study documents the occurrence of MDR bacteria carrying resistance and virulence-associated determinants in a captive *C. alba*, highlighting the value of genomic surveillance to improve our understanding of the distribution of clinically relevant bacterial isolates across different ecological settings.

**Supplementary Information:**

The online version contains supplementary material available at https://doi.org/10.1007/s11259-026-11490-z.

## Background

Animals serve as important biological indicators across diverse ecosystems. While commercial avian species have been the primary focus of microbiome research, ornamental and wild birds also play a crucial epidemiological role in the dissemination of infectious agents, antimicrobial-resistant bacteria, and resistance genes (Esposito et al. 2024; Gwenzi et al. 2021). Antimicrobial resistance (AMR) is a major challenge in both human and veterinary medicine, particularly due to the emergence and dissemination of multidrug-resistant (MDR) bacteria (Gwenzi et al. 2021; Magiorakos et al. 2012).

Captive avian species can serve as reservoirs for clinically significant bacterial pathogens and antimicrobial resistance determinants. Elevated population densities and stress levels increase susceptibility to infection and facilitate both direct and indirect zoonotic transmission (Ebani et al. 2021).

Among Gram-negative bacteria, members of the order Enterobacterales are of particular concern due to their ability to acquire and disseminate AMR determinants (World Health Organization 2024). *Escherichia coli* is frequently isolated from avian species and includes pathogenic isolates associated with respiratory, systemic, and reproductive diseases (Christensen et al. 2021; Kathayat et al. 2021; Nowazek et al. 2021). Similarly, *Enterobacter hormaechei*, a member of the *E. cloacae* complex, has emerged as an important opportunistic pathogen characterized by its capacity to accumulate resistance mechanisms and cause infections in both humans and animals (Han et al. 2023; Liu et al. 2022).

In contrast, *Cupriavidus* spp. is an environmental non-fermenting Gram-negative bacterium that has increasingly been reported as an opportunistic pathogen (Cuadrado et al. 2010; Papageorgiou et al. 2024). Although infections caused by *Cupriavidus* spp. are relatively uncommon, they have been associated with infections in humans and animals and may harbor clinically relevant resistance determinants (Kobayashi et al. 2016; Papageorgiou et al. 2024).

This study characterized *E. coli*, *E. hormaechei*, and *C. gilardii* isolated from the lungs and encephalon of a captive *Cacatua alba* that died at a conservation center using microbiological, phenotypic, genomic, and phylogenomic analyses.

## Case presentation

A 6-year-old female *C. alba* housed at a conservation center in Belo Horizonte, Minas Gerais, Brazil, was discovered deceased in an outdoor enclosure shared with psittacine birds of the genus *Amazona*. The aviary permitted occasional contact with free-living birds. A comprehensive post-mortem examination, including macroscopic and histopathological evaluation, was conducted to determine the cause of death.

**Fig. 1:**
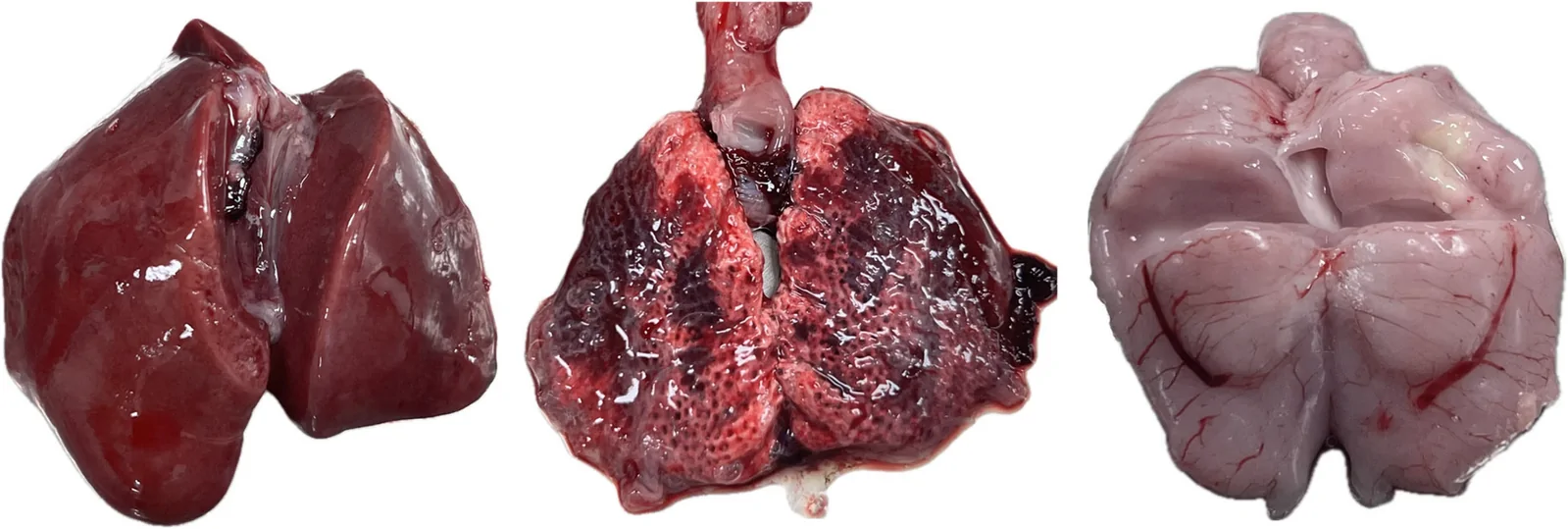
Post-mortem macroscopic findings in *C. alba*. (**A**) Enlarged liver (hepatomegaly). (**B**) Lungs with diffuse dark-red coloration. (**C**) Brain with a focal area of approximately 0.1 cm in diameter, yellowish, poorly delimited, with a depression on the cut surface

Gross examination identified an enlarged, yellow liver. The lungs appeared dark red and released a substantial quantity of foamy fluid upon sectioning. The encephalon exhibited a focal yellow area extending into the parenchyma (Fig. 1). Histopathological analysis demonstrated fibrinous, hemorrhagic, heterophilic pneumonia, while the liver displayed fibronecrotic heterophilic hepatitis. Despite the macroscopic abnormalities in the encephalon and repeated Gram staining of the tissue, no histopathological alterations were detected.

*E. coli* and *E. hormaechei* were isolated from the lungs, and *C. gilardii* (AV039) was recovered from the encephalon. Species identification was confirmed by matrix-assisted laser desorption/ionization time-of-flight mass spectrometry (MALDI-TOF MS).

Antimicrobial susceptibility testing was performed using the disk diffusion method on Mueller-Hinton agar with bacterial suspensions adjusted to the 0.5 McFarland standard (BrCAST, 2025). Multiple antimicrobial classes were evaluated. For *E. coli* and *E. hormaechei*, the following agents were tested: ampicillin, amoxicillin-clavulanic acid, ceftazidime, ceftriaxone, meropenem, imipenem, ertapenem, gentamicin, tobramycin, amikacin, ciprofloxacin, tetracycline, doxycycline, chloramphenicol, trimethoprim-sulfamethoxazole, and fosfomycin, with susceptibility interpreted according to the BrCAST interpretive criteria for Enterobacterales (BrCAST, 2025).

Species-specific clinical breakpoints are currently unavailable for *C. gilardii*. Consequently, disk diffusion inhibition zones were assessed using surrogate interpretive criteria from (BrCAST 2025) for *Burkholderia pseudomallei* as an exploratory reference. As these criteria have not been validated for *C. gilardii*, formal categorization of antimicrobial susceptibility was not feasible. Additionally, minimum inhibitory concentration (MIC) values were not determined, which precluded susceptibility characterization according to BrCAST recommendations (EUCAST, 2026). Therefore, the disk diffusion results are reported descriptively and should be interpreted with caution.

Both Enterobacterales isolates exhibited MDR phenotypes according to the international criteria proposed by Magiorakos et al. (2012). Both isolates remained susceptible to carbapenems. In contrast, the MDR classification was not applied to *C. gilardii* AV039 due to the absence of established interpretive criteria for several antimicrobial agents. Notably, this isolate demonstrated no inhibition zone for amoxicillin, amoxicillin-clavulanic acid, penicillin G, meropenem, gentamicin, amikacin, and rifampicin.

Genomic DNA was extracted and submitted to whole-genome sequencing using the Illumina HiSeq2500 platform with paired-end reads (2 × 150 bp). Sequence quality assessment, genome assembly, annotation, resistome characterization, virulome analysis, and comparative phylogenomic analyses were performed using established bioinformatic pipelines: FastQC (Andrew 2010), Trimmomatic (Bolger et al. 2014), CheckM2 (Chklovski et al. 2023), BUSCO (Manni et al. 2021), PanViTa (Rodrigues et al. 2023), Prokka (Seemann 2014), and Unicycler (Wick et al. 2017).

High-quality draft genome assemblies were obtained for all isolates, and assembly metrics are presented in supplementary material. The genomic data were deposited in the NCBI database under accession numbers AV037 (SAMN57124956; PRJNA1449518), AV038 (SAMN57124958; PRJNA1449519), and AV039 (SAMN57124957; PRJNA1449521).

Genomic analysis revealed that isolate AV037 harbored 52 resistance-associated genes, AV038 harbored 42, and AV039 harbored 8, as identified by the CARD gene count. However, most of these genes correspond to intrinsic resistance mechanisms, and only a subset is directly associated with an acquired antimicrobial resistance phenotype (Table 1). AV037 displayed greater resistome diversity, including 35 core genes. In contrast, isolate AV038 exhibited a more conserved resistome, with all 42 resistance-associated genes classified as core genes. AV039 contained eight core resistance-associated genes. No resistance-associated gene was shared among all three isolates.

**Table 1:** Distribution of antimicrobial resistance determinants and virulence-associated genes among bacterial isolates AV037, AV038, and AV039

Strain	Species	Phenotypic antimicrobial resistance profile	Antimicrobial resistance determinants	Virulence-associated genes
AV037	*E. coli*	Ampicillin, amoxicillin-clavulanic acid, ceftazidime, ceftriaxone, gentamicin, tobramycin, amikacin, ciprofloxacin	*-*	*flgD*, *csgC*, *spaQ*, *cfaA*, *cfaB*, *cfaC*, *cfaD/cfaE*, *espX1*, *espL4*, *espX4*, *espX5*, *pmrB*, *pmrA*, *fimG*, *fimF*, *fimC*, *fimI*, *fimA*, *fimE*, *fimB*, *allS*, *allA*, *allR*, *allB*, *allC*, *allD*, *clbQ*, *espR3*, *motB*, *fepE*, *fes*, *entD*, *ibeB*, *fdeC*, *ykgK/ecpR*, *yagZ/ecpA*, *yagY/ecpB*, *yagX/ecpC*, *yagW/ecpD*, *yagV/ecpE*, *lpfA*, *lpfC*, *aslA*, *ibeC*, *gspL*, *gspK*, *gspJ*, *gspI*, *gspH*, *gspG*, *gspF*, *gspE*, *gspD*, *gspC*, *espR1*, *hcp2/tssD2*, *tssB*, *vipB*, *fha*, *tssJ*, *tssL*, *hcp1/tssD1*, *rhs/PAAR*.
AV038	*E. hormaechei*	Ampicillin, amoxicillin-clavulanic acid, ceftriaxone, gentamicin, tobramycin, amikacin	*-*	*iroN*, *iroE*, *iroD*, *iroC*, *iroB*, *exeG*, *chuA*, *chuS*, *gtrA*, *gtrB*, *impA/tssA*, *icmF/tssM*, *clpV/tssH*, *hcp/tssD*, *dotU/tssL*, *vasE/tssK*, *vipB/tssC*, *vipA/tssB*, *iutA*, *hsiB1/vipA/tssB*, *STM0274*.
AV039	*C. gilardii*	Amoxicillin-clavulanic acid, meropenem*	*bla*_OXA−837_, *aac(3)-IVb*, *ant(3’’)-Ib*,* mcr-5.1*	*plc2*, *adeG*, *tufA*, *icl*.

*Results for *C. gilardii* should be interpreted with caution, as no species-specific clinical breakpoints are available. Disk diffusion inhibition zones were compared with the surrogate interpretive criteria for *B. pseudomallei* for exploratory purposes only; therefore, no formal susceptibility categorisation was assigned

The genes identified in this study were predominantly related to intrinsic efflux system components and their regulation (e.g., *acrA*, *acrB*, *acrD*, *acrE*, *acrF*, *mdtA*, *mdtB*, *mdtC*, *mdtE*, *mdtF*, *mdtG*, *mdtH*, *mdtK*, *emrA*, *emrB*, *mexB*, and *tolC*), global resistance regulators (e.g., *marA*, *ramA*, *sdiA*, *acrS*, *gadX*, *gadW*, *evgA*, *evgS*, *cpxA*, *kdpE*, *leuO*, *crp*, *hns*, and *rsmA*), outer membrane proteins (e.g., OmpA and OmpK37), and cell envelope determinants (e.g., *lptD*, *bacA*, *ugd*, *eptA*, *eptB*, and *pmrF*). Because the contribution of intrinsic genes to phenotypic resistance depends on gene expression and regulatory mechanisms, their presence alone should not be interpreted as evidence of phenotypic antimicrobial resistance. Antimicrobial resistance genes were identified in AV039 (e.g., *bla*_OXA−837_, *aac(3)-IVb*, *ant(3’’)-Ib*, *mcr-5.1*).

The pan-virulome analysis identified 160 virulence-associated genes across the three isolates: 135 in AV037, 91 in AV038, and 14 in AV039, with 65, 21, and 4 exclusive genes, respectively. The AV037 isolate was assigned to sequence type ST2541, while the *Enterobacter* isolate AV038 belonged to sequence type ST1597, according to the MLST scheme (Jolley et al. 2018).

**Fig. 2:**
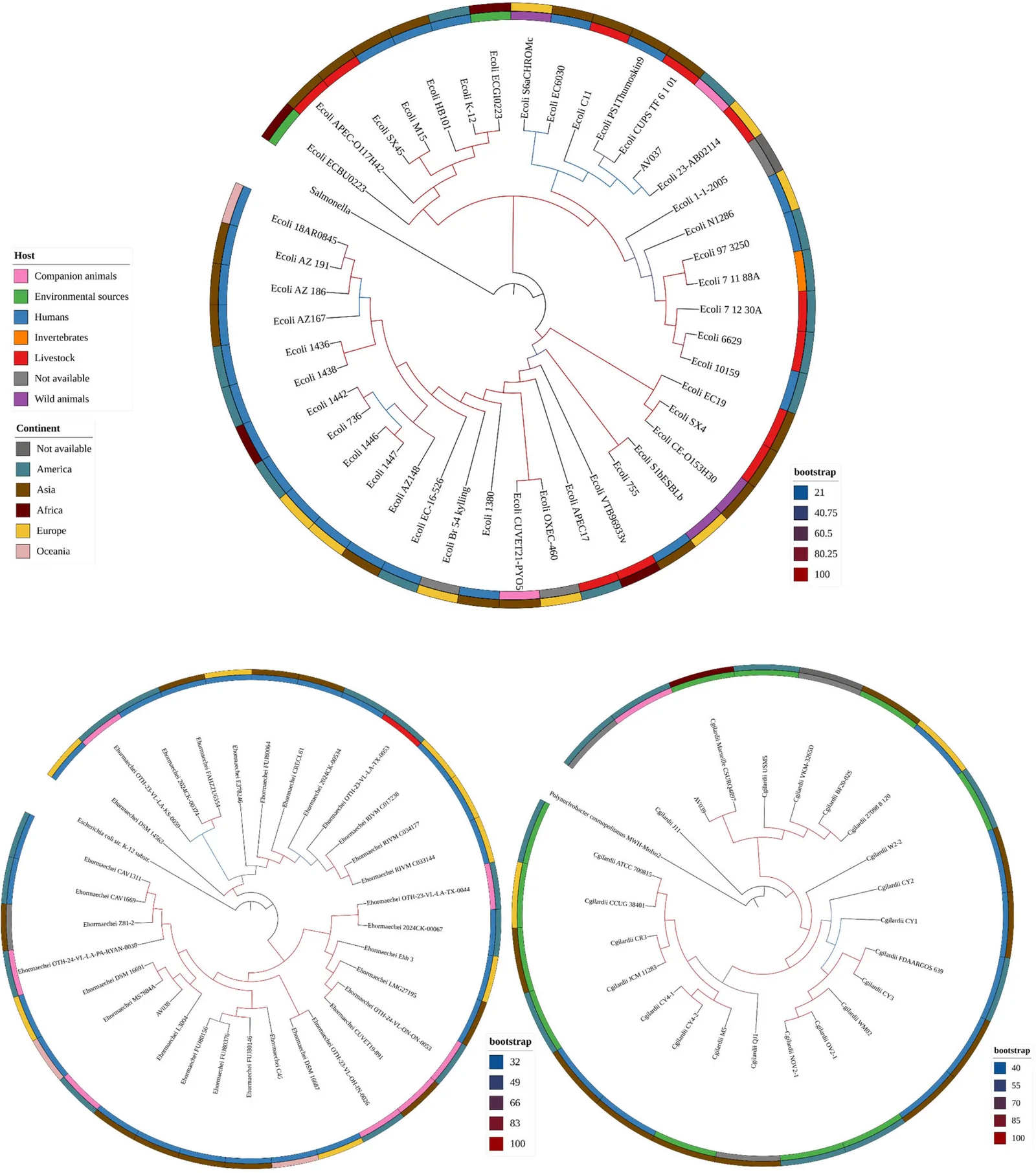
Phylogenomic tree constructed from whole-genome sequences using the maximum-likelihood method in OrthoFinder and visualized with iTOL. (**A**) *E. coli* (**B**) *E. hormaechei* (**C**) *C. gilardii*

To investigate the genetic relatedness of the isolates, comparative phylogenomic analyses were performed using publicly available genomes retrieved from the NCBI database. All genomes were initially filtered based on quality criteria (completeness ≥ 95% and contamination ≤ 5%) and selected according to host species and country of origin. Average nucleotide identity (ANI) was calculated using pyANI v0.2.12 with the ANIm (MUMmer) algorithm, and genomes sharing ≥ 95% ANI were considered to belong to the same species. No additional filtering based on sequence type (ST), host species, or geographic origin was applied to preserve the natural genetic diversity of the available dataset. Orthologous genes were identified using OrthoFinder v2.5.4 (Emms et al. 2019), and phylogenetic relationships were inferred using maximum-likelihood analysis in RAxML with 1,000 bootstrap replicates (Stamakis 2014). Phylogenetic trees were visualized using Interactive Tree of Life (iTOL) (Letunic 2024) (Fig. 2).

The dataset comprised 44 genomes for AV037 (human *n* = 22, animal *n* = 17, environmental *n* = 2, not available = 3), originating from Asia (*n* = 18), the Americas (*n* = 12), Europe (*n* = 8), Africa (*n* = 4), Oceania (*n* = 1) and not available (*n* = 1). For AV038, 32 genomes were included (human *n* = 23, animal *n* = 8, unidentified *n* = 1), distributed across Asia (*n* = 10), the Americas (*n* = 12), Europe (*n* = 8), and Oceania (*n* = 2). For AV039, 23 genomes were analyzed (environmental *n* = 11, human *n* = 8, animal *n* = 1, unidentified *n* = 3), distributed across Asia (*n* = 10), the Americas (*n* = 9), Europe (*n* = 2), Africa (*n* = 1), and one genome with unknown origin.

## Discussion

This report describes the recovery and characterization of *E. coli*, *E. hormaechei*, and *C. gilardii* from a captive *C. alba* submitted for post-mortem examination. Histopathological findings demonstrated severe fibrinous hemorrhagic heterophilic pneumonia and fibrinonecrotic heterophilic hepatitis, supporting a bacterial infectious process. *E. coli* and *E. hormaechei* were isolated from pulmonary tissue, whereas *C. gilardii* was recovered from the encephalon.

*E. coli* and *E. hormaechei* are recognized opportunistic and zoonotic pathogens frequently associated with MDR infections (Esposito et al. 2024; Han et al. 2023; Liu et al. 2022). In contrast, *C. gilardii* is primarily considered an environmental organism, but it has increasingly been reported as an opportunistic pathogen in immunocompromised individuals (Cuadrado et al. 2010; Kobayashi et al. 2016). The concurrent detection of these species in a single captive bird reinforces the potential role of ornamental birds as reservoirs of clinically relevant MDR bacteria within a One Health context.

According to the international criteria established by Magiorakos et al. (2012), isolates are classified as MDR if they are non-susceptible (resistant or intermediate) to at least one antimicrobial agent in three or more antimicrobial categories. Applying these criteria, both AV037 and AV038 were identified as MDR, exhibiting non-susceptibility in four and three antimicrobial categories: penicillins (ampicillin and amoxicillin), third-generation cephalosporins (ceftazidime and/or ceftriaxone), aminoglycosides (gentamicin, tobramycin, and amikacin), and fluoroquinolones (ciprofloxacin, levofloxacin, ofloxacin, or norfloxacin). In the case of *C. gilardii*, the lack of established interpretive criteria for several antimicrobial agents prevented a reliable MDR classification.

Previous studies have linked avian pathogenic *E. coli* (APEC) to severe respiratory disease in birds (Antão et al. 2008). Similarly, members of the *Enterobacter cloacae* complex, including *E. hormaechei*, have been associated with pneumonia and other invasive infections in both humans and animals (Hennigs et al. 2011). These findings are consistent with the present case, in which *E. coli* and *E. hormaechei* were isolated from lungs exhibiting severe inflammatory lesions. Although *C. gilardii* was isolated from the encephalon, no histopathological lesions were detected, and its clinical significance in this organ remains unclear.

*E. coli* ST2541 has been previously reported in studies involving both healthy and diseased companion animals in Brazil (Melo et al. 2018), where it was identified as one of the predominant sequence types circulating in dogs and cats. Furthermore, Walas et al. (2023) identified the same clonal group in isolates from both human and canine sources, indicating its circulation among different hosts within a One Health framework (Toombs-Ruane et al. 2020). In contrast, *E. hormaechei* ST1597 has been reported less frequently in the literature.

Phylogenomic analysis of AV037 revealed close phylogenetic relationships with strains isolated from diverse sources, such as calf cecal content (23-AB02114), swan feces in Ireland (S6aCHROMc), and pork from Asia (CUPS TF 6 1 01). These results suggest that AV037 shares a broad evolutionary background, aligning with genetically diverse *E. coli* isolates from various hosts and geographic regions. This observation underscores the recognized genomic plasticity of the species. Similarly, AV038 was grouped within a cluster of human-derived strains from Europe and Oceania (DSM 16691 and MS7884A), indicating shared evolutionary ancestry rather than recent epidemiological connection.

In contrast, AV039 clustered with environmental and human-associated isolates, including CSURQ4897, recovered from water and carrying the *mcr-5* gene, as well as strains isolated from plants and other environmental sources. This clustering reflects the limited but diverse genomic background available for *C. gilardii*. Although *C. gilardii* has traditionally been considered an environmental species, its placement within this phylogenetic context is consistent with previous reports describing its occurrence in environmental settings (Cuadrado et al. 2010; Kobayashi et al. 2016; Papageorgiou et al. 2024).

In the evaluated isolates, efflux pumps were the main mechanism identified. Among the five main efflux families, RND was predominant. The *acrB*,* eclo_acrA*,* acrD*,* acrF*,* acrE*, and *acrS* genes were mostly shared by AV037 and AV038 (Ferrari et al. 2013; Piddock 2006; Rosenberg et al. 2000).

Regulatory genes (*hns* and *sdiA*), efflux-related genes (*mdfA*,* emr*,* mdtM*,* mdtK*,* mdtA*, and *mdtG*), and outer membrane proteins (OmpA and OmpX) were identified in AV037 and AV038. These genetic elements have been associated with AMR, biofilm formation, bacterial adaptation, and host colonization, however, their presence alone is insufficient to infer a resistance phenotype (Confer et al. 2013; Edgar et al. 1997; Hai et al. 2024; Holdsworth et al. 2013; Lin et al. 2017; Perreten et al. 2001; Rahmati et al. 2002). Additional genes detected included *yojI*, *fosA*, *evgA*, *msbA*, and *bacA*, which have been associated to resistance against fosfomycin, macrolides, bacitracin, and other antimicrobial compounds but their contribution to the observed resistance phenotype requires further genomic and functional analyses (Delgado et al. 2005; Nishino et al. 2002; Woebking et al. 2005; Zhang et al. 2023).

Collectively, these findings highlight the diversity of resistance-associated genes identified in the isolates. Many of the detected genes, particularly those encoding efflux pump components (*acrB*,* acrA*,* acrD*,* acrF*) and regulatory genes such as *hns*,* sdiA*,* and evgA* (Rahmati et al. 2002; Hai et al. 2024), are intrinsic to the Enterobacterales genome and do not necessarily confer phenotypic resistance in isolation. In contrast, the *C. gilardii* isolate exhibited a smaller resistome, including the *mexB* gene, which has been previously associated with fluoroquinolone resistance in *Pseudomonas aeruginosa* (Niga et al. 2013). However, the contribution of this gene to the observed phenotype cannot be determined solely based on its presence. Isolate AV039 also harbored four major resistance determinants: *bla*_OXA−837_ (β-lactam resistance, particularly ampicillin), *aac(3)-IVb* (gentamicin and tobramycin resistance), *ant(3’’)-Ib* (spectinomycin and streptomycin resistance), and *mcr-5.1* (colistin resistance) (Ruiz et al. 2019).

AV037 and AV038 exhibited resistance to multiple β-lactams and aminoglycosides, yet remained susceptible to carbapenems. Both isolates also demonstrated resistance to fluoroquinolones, macrolides, lincosamides, and tetracyclines. These resistance phenotypes are likely associated with the presence of various intrinsic and acquired AMR determinants, including components of the AcrAB-TolC efflux system and related regulatory genes, which have previously been implicated in MDR in Enterobacterales (Ferrari et al. 2013; Piddock 2006; Rosenberg et al. 2000).

Virulence-associated genes were identified in all isolates, with a higher prevalence observed in *E. coli* and *E. hormaechei*. The majority of these genes were associated with adhesion, iron acquisition, motility, and secretion systems, which are recognized as key mechanisms for bacterial colonization and persistence (Haiko et al. 2013). *E. coli* isolated contained adhesion-associated genes (*lpfC*,* ibeC*, and *fimA*) and type VI secretion system components, consistent with previous reports in avian pathogenic *E. coli* (APEC) isolates from poultry (Stahlhofer et al. 2026). In comparison, *E. hormaechei* possessed iron acquisition-associated genes, such as *iutA*, which has been previously identified in APEC *E. coli* linked to colibacillosis, as well as the salmochelin operon (*iroBCDEN*), also detected in poultry farms in Brazil (da Silva et al. 2026).

The *C. gilardii* isolate harbored *plc2* and components of the AdeFGH efflux system, both of which are associated with environmental persistence and opportunistic pathogenicity (Xie et al. 2025). While the presence of these virulence determinants does not confirm their in vivo expression, their detection indicates a genetic potential for host colonization and pathogenicity. Because efflux-mediated resistance is contingent upon gene expression and regulatory activation, which were not assessed in this study, the specific contribution of these genes to the observed resistance phenotype remains unverified. In AV038, colonies observed within the fosfomycin inhibition zone suggested heteroresistance, however, further phenotypic testing is necessary to substantiate this finding (Campos et al. 2020).

In conclusion, this case demonstrates the presence of MDR bacterial species possessing significant resistance and virulence determinants in a single captive ornamental bird. The phylogenomic similarity among animal, human, and environmental isolates underscores the interconnectedness of AMR dissemination and highlights the necessity of surveillance strategies within a One Health framework. Additionally, this report contributes to the limited genomic data on bacterial pathogens isolated from ornamental avian species and underscores their potential epidemiological significance.

## Supplementary Information

Below is the link to the electronic supplementary material.

Supplementary Material 1

Supplementary Material 2

Supplementary Material 3

Supplementary Material 4

## Data Availability

No datasets were generated or analysed during the current study.
