# Supplementary material for: Genomic analysis of Escherichia coli, Enterobacter hormaechei, and Cupriavidus gilardii isolated from Cacatua alba
Source: Vet Res Commun. 2026 Aug 29;50(6):544. doi: 10.1007/s11259-026-11490-z (PMC13695599; doi:10.1007/s11259-026-11490-z)
Supplement: Supplementary file 1 [file 11259_2026_11490_MOESM1_ESM.docx]

1. Genome annotation of AV037, AV038, and AV039

| **Identification** | **Species** | **CDS** | **rRNA** | **tRNA** | **Genome size** |
| --- | --- | --- | --- | --- | --- |
| AV037 | *Escherichia coli* | 4535 | 6 | 84 | 4879329 |
| AV038 | *Enterobacter hormaechei* | 4370 | 3 | 69 | 4697962 |
| AV039 | *Cupriavidus gilardii* | 4724 | 3 | 57 | 5363983 |

2. Genomic sequencing quality of bacterial strains AV037, AV038, and AV039

| **ID** | **Species** | **Contigs** | **N50** | **L50** | **GC (%)** | **Completeness (%)** |
| --- | --- | --- | --- | --- | --- | --- |
| AV037 | *Escherichia coli* | 127 | 203.462 | 9 | 50,73 | 100 |
| AV038 | *Enterobacter hormaechei* | 32 | 403.202 | 5 | 55,65 | 100 |
| AV039 | *Cupriavidus gilardii* | 55 | 245.203 | 7 | 67,39 | 100 |

**3.** Antibiogram profile of *C. gilardii*.

| **Antimicrobial category** | **Antimicrobial** | **AV039**  ***C. gilardii*** | |
| --- | --- | --- | --- |
|  |  | **IZD (mm)** | **Interpretation** |
| Penicillins | Amoxicillin | 0 | NA |
|  | Amoxicillin-clavulanic acid | 0 | R |
|  | Penicillin G | 0 | NA |
| Cephalosporins | Cephalexin | 28 | NA |
|  | Cefotaxime | 23 | NA |
| Carbapenems | Meropenem | 0 | R |
| Aminoglycosides | Gentamicin | 0 | NA |
|  | Amikacin | 0 | NA |
| Fluoroquinolones | Norfloxacin | 26 | NA |
| Rifamycins | Rifampicin | 0 | NA |

IZD, inhibition zone diameter (mm); S, susceptible; I, intermediate; R, resistant; –, not performed; NA, not available (no inhibition zone data).

Resistance profile and components identified across the analyzed bacterial isolates.

| **Isolate** | **Species** | **Identified resistance-associated genes** |
| --- | --- | --- |
| **AV037** | *E. coli* | *acrB*, *rosB*, *rosA*, *kdpE*, *Ecol_mdfA*, *kpne_KpnF*, *kpne_kpnE*, *mdtH*, *mdtG*, *ompA*, *msbA*, *marA*, *kpne_ompK37*, *hns*, *rsmA*, *tolC*, *bacA*, *yojI*, *acrD*, *eptB*, *mdtC*, *mdtB*, *mdtA*, *ugd*, *mdtK*, *sdiA*, *pmrF*, *crp*, *lptD*, *acrF*, *acrE*, *emrB*, *emrR*, *cpxA*, *leuO*, *emrA*, *acrS*, *mdtP*, *mdtO*, *mdtN*, *eptA*, *EC-15*, *mdtM*, *Ecol_acrA*, *gadX*, *gadW*, *mdtF*, *mdtE*, *evgS*, *evgA*, *emrK*, *emrY*. |
| **AV038** | *E. hormaechei* | *acrB*, *Eclo_acrA*, *rosB*, *rosA*, *ramA*, *kdpE*, *Ecol_mdfA*, *kpne_KpnF*, *kpne_kpnE*, *mdtH*, *mdtG*, *ompA*, *msbA*, *marA*, *kpne_OmpK37*, *hns*, *rsmA*, *tolC*, *bacA*, *mdtQ*, *yojI*, *acrD*, *eptB*, *mdtC*, *mdtB*, *mdtA*, *ugd*, *mdtK*, *sdiA*, *bla*_ACT-35_, *fosA2*, *pmrF*, *arnT*, *crp*, *lptD*, *acrF*, *acrE*, *emrB*, *kpne_KpnG*, *emrR*, *cpxA*, *leuO* |
| **AV039** | *C. gilardii* | *mexB*, *ceoB*, *muxB*, *mdsB*, *bla*_OXA-837_, *aac(3)-IVb*, *ant(3'')-Ib, mcr-5.1* |

* Overview of all resistance-related genomic determinants identified by bioinformatic analysis. These data encompass intrinsic pathways, efflux pump genes, and global regulators, whose presence alone does not allow the phenotype to be inferred without demonstrated expression or functional alterations.
